# When Left Is Right and Right Is Wrong: A Case Report of Two Near-Miss Wrong-Sided Peripheral Nerve Blocks

**DOI:** 10.1155/2022/1541827

**Published:** 2022-06-15

**Authors:** Alexander M. DeLeon, Alexander G. Samworth, Bashar F. Kazanji

**Affiliations:** ^1^Northwestern Memorial Hospital, 251 East Huron Suite F5-704, Chicago, Illinois 60611, USA; ^2^Fellow in Regional Anesthesiology and Acute Pain Medicine, Northwestern Memorial Hospital, 251 East Huron Suite F5-704, Chicago, Illinois 60611, USA; ^3^Resident in Anesthesiology, Northwestern Memorial Hospital, 251 East Huron Suite F5-704, Chicago, Illinois 60611, USA

## Abstract

Wrong-sided peripheral nerve blocks occur with a surprisingly high frequency despite being described as a “never event.” Timeout procedures are performed and documented, yet timeout omission is rarely cited as a contributing factor for wrong-sided blocks. We present two cases of near-miss wrong-sided peripheral nerve blocks and provide recommendations based on the current literature and the most common contributing factors.

## 1. Introduction

Wrong-sided peripheral nerve blocks are considered a never event yet occur with a surprisingly high frequency [1]. The reported incidence of wrong-sided peripheral nerve blocks is as high as 7.5 per 10,000 cases, which is higher than the rate of anaphylaxis under general anesthesia (2.9 per 10,000 cases) [1, 2]. Despite numerous recommendations on preventing wrong-sided blocks, including timeout procedures and checklists, these never events still occur [1, 3, 4].

We present two cases of near-miss wrong-sided blocks to illustrate the pitfalls of the timeout process and make recommendations based on the most commonly reported causes of these events.

## 2. Case 1 Description

A 56-year-old woman presented for right-sided talonavicular subtalar fusion for a history of arthritis in the subtalar joint of her left foot. She had a medical history which included HTN and rheumatoid arthritis. She had no other significant medical history, and her medications included losartan and methotrexate.

Her weight was 73.8 kg, and her BMI was 29.8 kg/m^2^. Her vital signs were BP 140/84 mmHg, HR 101 bpm, and RR 18/min. The anesthetic plan consisted of a right-sided adductor canal block and a right-sided infragluteal sciatic nerve block, both under ultrasound guidance. The patient's hospital-issued sock was also removed from her surgical foot. The peripheral nerve blocks were performed in the preoperative area and complied with ASA standard monitoring [5].

After a procedural timeout was performed, which included confirming the patient's identity, surgical mark, and surgical consents, with the patient nonsedated in the supine position, the patient received 1 mg of midazolam and 50 mcg of fentanyl intravenously.

A right-sided adductor canal block was performed with the patient in the supine position; the ultrasound machine was located on the patient's left side during the performance of the block with the anesthesia provider positioned to the patient's right. After the adductor canal block was completed, the patient was positioned prone to perform the infragluteal sciatic nerve block. The ultrasound remained in the same position relative to the bed.

Both providers agreed to proceed with scanning what was believed to be the surgical limb. Due to difficulty obtaining visualization of the nerve, the second provider began to attempt to image the sciatic nerve. The second provider began to scan the leg and noticed that the sock was still visible on the patient's foot despite remembering it had been removed. The team realized they were scanning the left, nonsurgical leg and thus reversed course and placed the ultrasound on the opposite side of the room and performed the appropriate, right-sided sciatic nerve block. The patient underwent surgery uneventfully with intraoperative sedation and was discharged without incident.

## 3. Case 2 Description

A 41-year-old male presented for a right-sided open reduction and internal fixation (ORIF) of a Lisfranc fracture and tarsal-metatarsal fusion for a traumatic injury. His medical history was significant for receiving an ORIF of his left distal tibia to treat a different injury two weeks earlier. The patient's medical history also included alcohol abuse. The patient was not taking medications other than what he received for pain. His weight was 79.4 kg with a BMI of 25.84 kg/m^2^. His preoperative vital signs were BP 116/62 mmHg, HR 92 bpm, and RR 16/min. All lab values were within normal limits.

**Figure 1:**
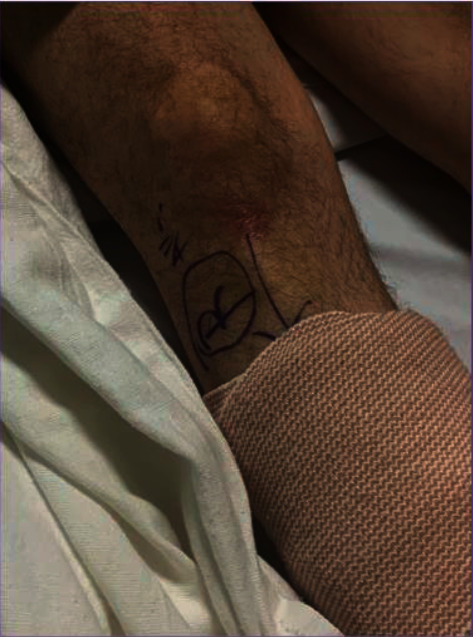
The surgical mark for Case 2 visible on the anterior portion of the patient's operative leg, and the mark was easily visible during the performance of the adductor canal block.

The surgical site mark was visible (Figure 1). The anesthetic plan was to perform a right-sided adductor canal block (supine position) followed by a right-sided infragluteal sciatic nerve block (prone position) in the preoperative area as described in Case 1. Both blocks were performed using ultrasound guidance. For this case specifically, the anesthesiologist placed a mark on the posterior aspect of the operative side to be visible during the performance of the infragluteal sciatic block. A timeout procedure confirmed the patient's name, surgical site, surgical mark presence (anterior and posterior), and consent. The patient was not sedated before the timeout. After the timeout was performed, the patient was administered 1 mg of midazolam and 50 mcg of fentanyl. The adductor canal block was performed with the patient in the supine position and the ultrasound machine to the patient's left (similar to Case 1). The resident was located to the patient's right.

**Figure 2:**
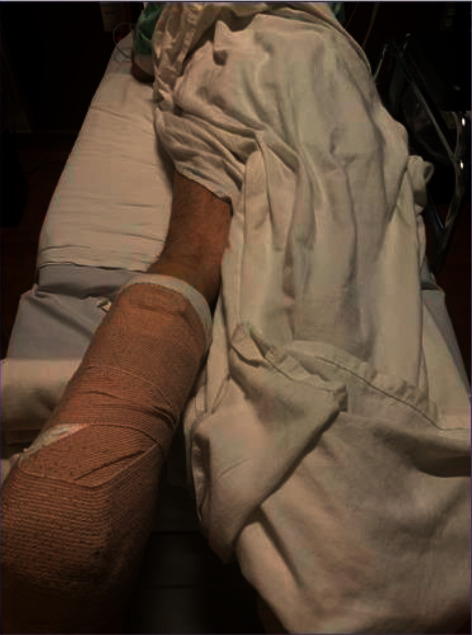
The patient was repositioned supine. The procedural mark was not visualized during the pause point because the team began to prepare the opposite (left, nonsurgical) leg (Case 2).

**Figure 3:**
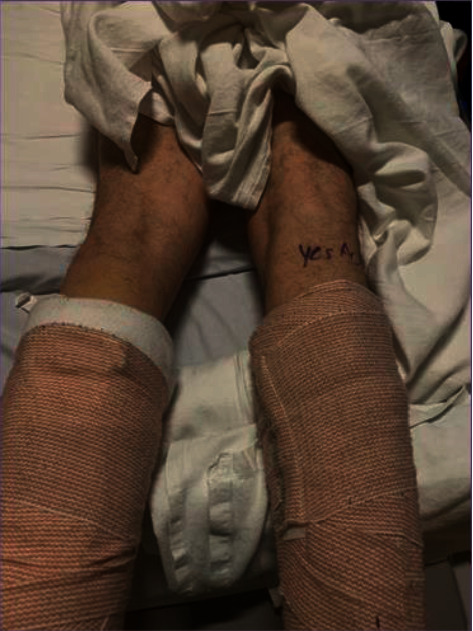
The near-miss was prevented from becoming a wrong-sided block by correctly visualizing the procedural mark on the right (i.e., correct) limb as part of the pause point before the second block (Case 2).

For the sciatic block, the patient was repositioned to the prone position. Before scanning the patient's leg, a pause point was initiated to observe the separate anesthesia procedural marks (Figure 2). A mark was not observed on the leg prepared for imaging. The second provider uncovered the opposite leg and visualized the correct surgical limb mark (Figure 3). The nerve block then proceeded on the correct, right-sided limb, and the surgery and recovery followed without incident.

## 4. Discussion

The two cases presented in this case report involved the same anesthesiologist. Placing a procedural mark on the second case directly resulted from the near-miss case described in this report first. Deutsch et al. reviewed 70 publications searching for mentions of various causes of wrong-sided blocks [4]. Time pressure was mentioned most as the single most common cause associated with wrong-sided procedures. The realm of regional anesthesia is a somewhat unique area in healthcare where the clinicians are pressured to complete a process by a particular timepoint. Delays in first case starts are a commonly measured outcome [6]. An anesthesia team performing a nerve block outside of the operating room, unable to complete a nerve block before the starting time of surgery, could be penalized for the delay. Thus, the production pressure for the first start completion of peripheral nerve blocks can be excessive.

Deutsche et al. also mentioned the cause of “mark not visible” as one of the most commonly cited contributory factors. Also, “patient repositioned, including front/back,” was one of the most common factors [4]. Of note, lack of timeout was one of the least mentioned contributing factors by as described by Deutsche et al. Our cases presented in this report were appropriately timed out before proceeding, yet both were still near-misses.

Another contributory factor to wrong-sided blocks is an incorrect environmental cue [4]. In Case 1, one provider preferred the ultrasound machine on the patient's right and the other preferred it to the left (opposite side of the patient) when performing a sciatic nerve block on the right leg. Thus, the environmental cue of ultrasound positioning likely misled the anesthesia provider toward the opposite, nonsurgical side. In Case 2, the splint/cast on the nonoperative leg was an incorrect environmental cue that initially misled the anesthesia team.

For this case report, the author, AD, changed his practice after the first case to address potential contributing factors. The practices changed were to alert residents that they “do not feel pressure to complete this block on time, and I would prefer us to delay surgery over providing suboptimal care.” Another practice change was to mark the site of the nerve block and not only the site of surgery. A nerve block was performed anteriorly and posteriorly in both the presented cases. Thus, a second mark was placed on the posterior leg of Case 2 (Figure 3). The final change was to establish a pause point before performing the second block when two blocks are performed. Some recommend a separate timeout for each procedure, yet a single pause point confirming the side and visualizing the second mark may have a greater compliance rate [3]. During the initial timeout, both anterior and posterior marks should be visualized.

The quality improvement literature is moving away from focusing on blaming individuals and more toward improving processes. Educating practitioners that they need to “always perform a timeout” may be a low-yield practice given the rarity of reported failure to perform timeouts in the wrong-sided block reports [4]. The cases presented in this report illustrate various known contributory factors to wrong-sided blocks, including marks not visible, patient repositioned, incorrect environmental cues, and inadequate communication. One factor that did not contribute to our cases was the omission of the timeout procedure. The pause point and procedure mark for the prone nerve block, separate from the anterior mark, provided the final check, which may have prevented the second case from receiving a wrong-sided block. The visible mark at the time of nerve block is reassuring. It can be checked multiple times when performing the nerve block before scanning, needle insertion, and immediately before local anesthetic injection. The simple practice of labeling both sides of the leg when two blocks are performed and confirming the second mark after repositioning can potentially address the most commonly cited contributing factors to wrong-sided block procedures.

### 4.1. Author Recommendations

- Reduce time pressure to perform regional blocks by stressing the importance of quality over on-time starts when nerve blocks are involved
- Be aware of environmental cues by making mental notes of patients with bilateral injuries
- Visualize the procedure mark immediately prior to performing nerve block. Occasionally, a separate block mark may be needed if surgical mark is far from the nerve block site.
- Place separate procedure marks when multiple blocks (i.e., front/back blocks) are performed on the same patient. Visualize both marks during the initial timeout.
- After repositioning, institute a pause point to confirm that the second procedural mark is visible before performing the second block

## Data Availability

The data used to support this study are included within the article.
